# Characterization of the inflammatory proteome of synovial fluid from patients with psoriatic arthritis: Potential treatment targets

**DOI:** 10.3389/fimmu.2023.1133435

**Published:** 2023-03-22

**Authors:** Nuria Barbarroja, Maria Dolores López-Montilla, Laura Cuesta-López, Carlos Pérez-Sánchez, Miriam Ruiz-Ponce, Clementina López-Medina, Maria Lourdes Ladehesa-Pineda, Chary López-Pedrera, Alejandro Escudero-Contreras, Eduardo Collantes-Estévez, Iván Arias-de la Rosa

**Affiliations:** ^1^ Rheumatology Service/Department of Medical and Surgical Sciences, Maimonides Institute for Research in Biomedicine of Cordoba (IMIBIC), Reina Sofia University Hospital, University of Cordoba, Córdoba, Spain; ^2^ Cobiomic Bioscience S.L, Cordoba, Spain

**Keywords:** synovial fluid (SF), psoriatic arthritis, inflammation, proteome, proximity extension assay (PEA)

## Abstract

**Objectives:**

1) To characterize the inflammatory proteome of synovial fluid (SF) from patients with Psoriatic Arthritis (PsA) using a high-quality throughput proteomic platform, and 2) to evaluate its potential to stratify patients according to clinical features.

**Methods:**

Inflammatory proteome profile of SF from thirteen PsA patients with active knee arthritis were analyzed using proximity extension assay (PEA) technology (Olink Target 96 Inflammation panel). Four patients with OA were included as control group.

**Results:**

Seventy-nine inflammation-related proteins were detected in SF from PsA patients (SF-PsA). Unsupervised analyzes of the molecular proteome profile in SF-PsA identified two specific phenotypes characterized by higher or lower levels of inflammation-related proteins. Clinically, SF-PsA with higher levels of inflammatory proteins also showed increased systemic inflammation and altered glucose and lipid metabolisms. Besides, SF from PsA patients showed 39 out of 79 proteins significantly altered compared to SF-OA specifically related to cell migration and inflammatory response. Among these, molecules such as TNFα, IL-17A, IL-6, IL-10, IL-8, ENRAGE, CCL20, TNFSF-14, OSM, IFNγ, MCP-3, CXCL-11, MCP4, CASP-8, CXCL-6, CD-6, ADA, CXCL-10, TNFβ and IL-7 showed the most significantly change.

**Conclusion:**

This is the first study that characterizes the inflammatory landscape of synovial fluid of PsA patients by analyzing a panel of 92 inflammatory proteins using PEA technology. Novel SF proteins have been described as potential pathogenic molecules involved in the pathogenesis of PsA. Despite the flare, inflammatory proteome could distinguish two different phenotypes related to systemic inflammation and lipid and glucose alterations.

## 1 Introduction

Psoriatic arthritis (PsA) is a heterogenous inflammatory joint disease that includes psoriasis, onychopathy, peripheral or axial clinical symptoms, and a high number of comorbidities. Synovial membrane inflammation, driven by effector T-cell activation and altered inflammatory cytokine expression, is a key feature of this disease (1, 2).

The recent development of the proximity extension assay (PEA) technique can contribute to identifying novel protein biomarkers associated with PsA and to improving the biological understanding and the future management of PsA, leading to novel intervention targets (3). Using this breakthrough technology, we have previously identified some cardiovascular (CV) related proteins associated with clinical characteristics of PsA patients highlighting the association between inflammation and cardiovascular disease comorbidity. Besides, we suggested the potential role of circulating CV disease-related proteins as biomarkers of insulin resistance, disease activity, and non-response to therapy in PsA (4).

The PEA consists of a dual-recognition immunoassay, where two specific antibodies labeled with DNA oligonucleotides simultaneously bind to a target protein. This leads the two antibodies to converge and their DNA oligonucleotides to hybridize, serving as a template for a DNA polymerase-dependent extension step. This generates a double-stranded DNA which is unique for the specific protein. After the hybridization and extension, occurs PCR amplification, where the concentration of the PCR product is directly proportional to the initial concentration of the target protein.

To the best of our knowledge, this is the first study that characterizes the inflammatory landscape of synovial fluid (SF) of PsA patients by analyzing a panel of 92 inflammatory proteins using PEA technology (Olink Proteomics). Thus, we aimed to delineate the inflammatory proteome of SF from PsA patients, to identify different phenotypes of PsA patients by unsupervised cluster analysis of the inflammatory proteome, and finally to compare the levels of these inflammatory proteins with SF of osteoarthritis (OA) patients as a control group.

## 2 Patients and methods

In this study, thirteen PsA patients (mean age 51.4±10.7 years, 6 females and 7 males) with active knee arthritis were included. PsA patients were diagnosed by a rheumatology specialist following the classification for psoriatic arthritis (CASPAR) criteria (5). The diagnosis of active inflammatory knee arthritis was performed through levels of c-reactive protein and physical examination by the rheumatologist. SF samples were collected from patients at the Rheumatology department of Reina Sofia Hospital of Cordoba, Spain, after informed consent. The study was approved by the Ethical Committee of the Reina Hospital of Cordoba. SF samples were centrifuged at 3000g for 10 min and the supernatant was diluted four times and stored at -80°C. Taking into account the flare state of the PsA patients confirmed by the specialist, we next selected the Olink inflammation target 96 panel (Cobiomic Bioscience, S.L) to perform Proximity Extension Assay (PEA) analysis of the levels of 92 proteins that are primarily associated with immune and inflammatory biological processes.

Specifically, PsA patients were characterized by the presence of high rates of inflammation (mean C-reactive protein 32.2 ± 10.6 mg/dL). Eight patients showed peripheral form and five patients presented mixed form (peripheral plus sacroiliitis). Regarding the treatment, eleven patients were treated with non-steroidal anti-inflammatory drugs, 3 with corticosteroids, 9 with methotrexate, 3 with leflunomide, 2 with apremilast, and 3 with biologicals (2 with ustekinumab and 1 with golimumab). Four patients with OA were included.

## 3 Results

**Figure 1:**
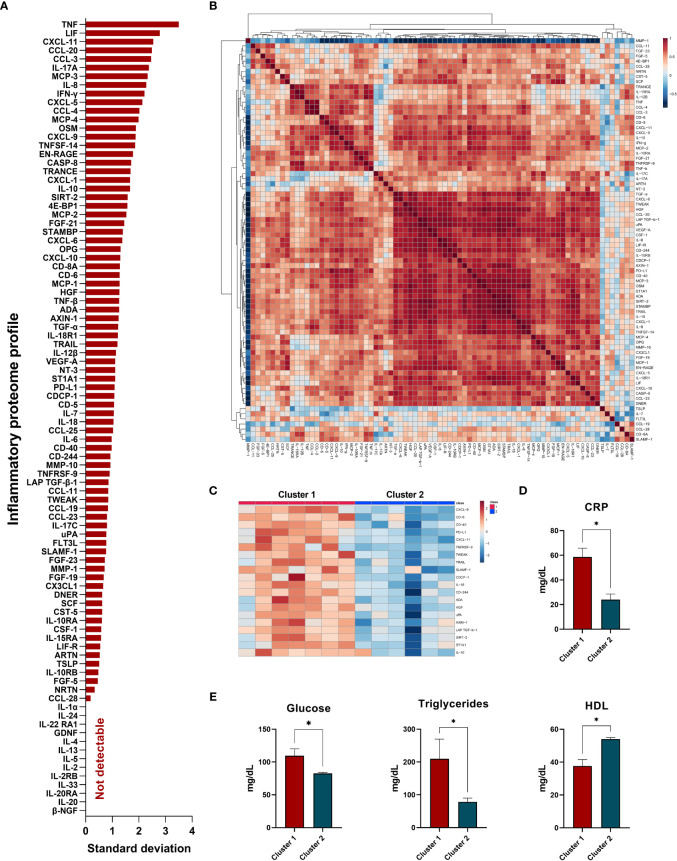
Inflammatory proteome profile in synovial fluid from PsA patients: cluster analysis and association studies with clinical parameters. **(A)** Standard deviation of proteins detected in synovial fluid. **(B)** Heatmap correlation of detected proteins. **(C)** Heatmap of top inflammatory proteins which differentiate PsA clusters. **(D)** Levels of C-reactive protein (CRP) among clusters. **(E)** Levels of glucose, triglycerides, and high-density lipoprotein (HDL)-cholesterol among clusters. *Significant differences between cluster 1 *vs* cluster 2 (p<0.05).

Amongst the 92 inflammatory proteins, 13 proteins had levels below the lower limit of detection (LLOD) which were discarded from further analysis. Thus 79 inflammatory proteins were detectable. **Figure 1A** shows the average amount of variability (standard deviation) of the protein levels in our cohort of PsA patients, being the top ten variable molecules: tumor necrosis factor alpha (TNFα), leukemia inhibitor factor (LIF), C-X-C motif chemokine ligand 11 (CXCL-11), C-C motif chemokine ligand 20 (CCL-20), C-C motif chemokine ligand 3 (CCL-3), interleukin 17A (IL-17A), monocyte chemoattractant protein 3 (MCP-3), interleukin 8 (IL-8), interferon gamma (IFNγ) and C-X-C motif chemokine ligand- 5 (CXCL-5). Accordingly, the levels of some of these inflammatory molecules were highly correlated, showing a strong relationship between them (**Figure 1B**, heatmap of Pearson correlation between the levels of the 79 proteins).

In terms of PsA clinical forms, our results showed that levels of AXIN1 were significantly elevated in SF-PsA patients with peripheral forms compared to those with mixed forms (mean 3.53 ± 0.93 *vs* 4.73 ± 0.91, *p*-value = 0.05). Similarly, IL-33 levels were also significantly increased in peripheral forms compared to mixed forms (mean 0.47 ± 0.21 *vs* 0.98 ± 0.19, *p*-value = 0.003).

Interestingly, unsupervised cluster analysis identified two clusters of PsA patients with distinctive molecular patterns. The most contributing proteins to discriminate clusters of PsA patients were C-X-C motif chemokine ligand 9 (CXCL-9), T-cell differentiation antigen (CD-6), cluster differentiation molecule 40 (CD-40), programmed cell death 1 ligand 1 (PD-L1), C-X-C motif chemokine ligand 11 (CXCL-11), TNF receptor superfamily member 9 (TNFRSF-9), TNF-related weak inducer of apoptosis (TWEAK), TNF-related apoptosis inducing ligand (TRAIL), signaling lymphocytic activation molecule (SLAM-1), CUB domain-containing protein 1 (CDCP-1), interleukin-18 (IL-18), natural killer cell receptor 2B4 (CD-244), adenosine deaminase (ADA), hepatocyte growth factor (HGF), urokinase-type plasminogen activator (uPA), axin-1 (AXIN-1), latency-associated peptide and transforming growth factor beta-1(LAP-TGFb1), NAD-dependent protein deacetylase sirtuin-2 (SIRT-2), sulfotransferase 1A1 (ST1A1) and interleukin 10 (IL-10) (**Figure 1C**).

Thus, PsA patients from cluster 1 presented significantly higher levels of these inflammatory proteins than cluster 2, revealing a specific group of patients with high inflammatory molecular pattern in SF (**Figure 1D**). Clinically, PsA patients from cluster 1 showed higher levels of CRP, glucose, and triglycerides and lower levels of HDL compared to cluster 2, demonstrating the association of the imbalance in lipid profile with the inflammatory burden in PsA (**Figure 1E**).

**Figure 2:**
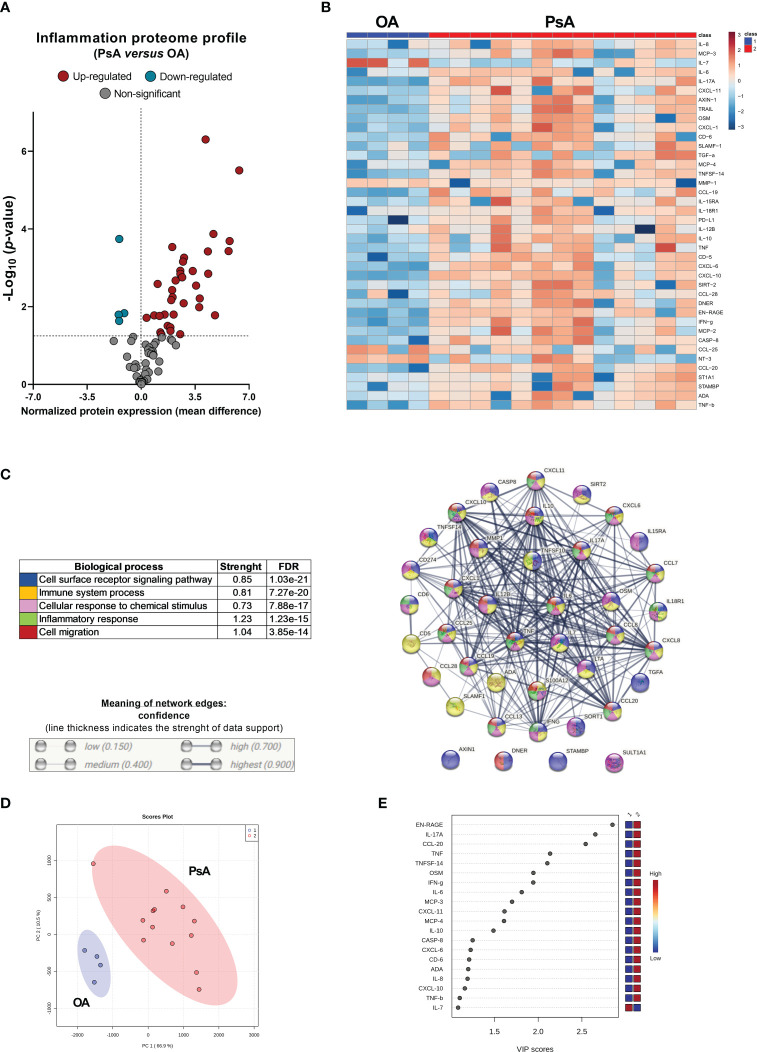
PsA synovial fluid show a significant alteration of the inflammatory proteome in relation to osteoarthritis (OA). **(A)** Volcano plot of 92 inflammation-related proteins in PsA compared to OA. **(B)** Heatmap of significant proteins altered in PsA compared to OA. **(C)** Functional classification of the altered levels of the inflammatory proteome in PsA by STRING platform. **(D)** Principal component analysis (PCA) summarizing the differences in the molecular profile of each group. **(E)** The most contributing proteins to discriminate PsA compared to OA patients.

Compared to OA patients, SF from PsA patients showed 39 proteins significantly altered, 35 upregulated and 4 downregulated (**Figure 2A**). Interestingly, IL17A was not detectable in the SF from OA patients (levels below the LLOD). Principal component analysis (PCA) confirmed the separation of specific molecular profiles between PsA and OA (**Figure 2B**). Enrichment analysis using the STRING platform (version 11.5, STRING CONSORTIUM 2022) identified the biological functions altered in SF from PsA compared to OA patients, including cell surface receptor signaling pathway, immune system process, cellular response to chemical stimulus, inflammatory response and cell migration (**Figure 2C**).

Likewise, hierarchical cluster analysis revealed two clusters differentiating the two groups of patients (OA and PsA) (**Figure 2D**). The most contributing proteins to differentiate PsA from OA patients were C-C motif chemokine ligand (CCL-20), IL-17A, ENRAGE (S100A12), tumor necrosis factor superfamily member 14 (TNFSF-14), C-C motif chemokine ligand 3 (CCL-3), IFN-γ, uPA, TNF, caspase-8 (CASP-8), macrophage/monocyte chemotactic protein 4 (MCP-4), IL-8, interleukin 6 (IL-6), matrix metalloproteinase-1 (MMP-1), macrophage/monocyte chemotactic protein 3 (MCP-3) and TNF-β (**Figure 2E**).

## 4 Discussion

Infiltrating immune cells in the synovial membrane of PsA patients produce proinflammatory mediators that activate fibroblast-like synoviocytes, which then invade adjacent cartilage and bone. In this sense, key cytokines have been described to have an important role in the synovium in PsA settings. Among them, TNFα, IL-23, IL-17A/F, IL-22, IL-6, and IL-10, produced by macrophages, T cells, fibroblast-like synoviocytes or B cells have been shown increased in the synovial fluid and synovial tissue and are responsible for the stimulation of pathogenic T-cells and the activation of osteoclasts, chondrocytes and fibroblast-like synoviocytes (2, 6–9).

Nowadays it is fully recognized that the proteome can provide a deeper understanding of the pathophysiology of PsA, a key source for detecting new biomarkers. PEA analysis, developed by Olink Proteomics, is a powerful technique for identifying and measuring multiple biomarkers simultaneously in a single sample.

Previously, label-free quantitative proteomics using liquid chromatography coupled to mass spectrometry (LC-MS/MS) were used to identify differently expressed proteins in SF from PsA patients compared to SF-OA. The procedure required previous pre-analytical sample processing, high-performance liquid chromatography (HPLC) using strong cation exchange (SCX), LC-MS/MS and protein identification and quantification. Interestingly, they identified 137 proteins differentially expressed among PsA and OA associated with acute-phase response signaling, granulocyte adhesion and diapedesis, and production of nitric oxide and reactive oxygen species in macrophages. However, lower repeatability, which may compromise the identification of smaller quantitative differences between samples is one of the potential disadvantages in this study (10). In this sense, PEA analysis provides several advantages over traditional biomarker analysis methods. First, it can detect multiple biomarkers simultaneously, providing a comprehensive view of the biological processes involved in a disease or condition. Second, it has a high degree of sensitivity and specificity, allowing for the detection of low-abundance biomarkers in small samples. Third, it requires minimal sample preparation and handling, making it a quick and efficient method for biomarker analysis. However, despite its high potential and its increasing use in proteomic research and biomarker identification, the Olink technology has been scarcely utilized in the field of psoriatic arthritis.

We have recently performed PEA analysis in plasma of a hundred PsA patients in the search of potential CVD novel biomarkers related to clinical features of the disease such as body surface area affected by psoriasis (Pso), the presence of onychopathy, disease activity, systemic inflammation and cardiometabolic comorbidities, such as insulin resistance or obesity. Besides, potential biomarkers of non-response to therapy with methotrexate or apremilast were also defined (4). In addition, *Leijten E and coworkers* demonstrated a serum proteomic signature shared by both PsA and Pso patients using 11 different Olink panels: cardiometabolic, cardiovascular II and III, cell regulation, development, immune response, inflammation, metabolism, neurology, oncology II and organ damage. They conclude that PsA and Pso patients are quite difficult to discriminate by this proteomic approach and highlight the study of synovial fluid in the future. Among others, they described levels of IL6 significantly elevated in PsA and correlated with disease activity (11). In our study, IL6 was one of the most contributing proteins to differentiate SF from PsA to OA patients.

Herein, our exploratory study shows for the first time the technical performance of this assay on synovial fluid from PsA and OA patients. Using this high-quality throughput proteomic platform validated for its use on synovial fluid samples (12) we identified 79 novel inflammatory molecules present in the synovial fluid of PsA patients. Importantly, despite the joint inflammation, we detected two clusters of PsA patients depending on the levels of 20 inflammatory-related proteins in SF, cluster 1 of PsA patients with a strong inflammatory pattern associated with systemic inflammation based on circulating CRP levels and different levels of glucose and lipids in cluster 1 compared to cluster 2, suggesting the link between SF inflammatory proteome with metabolic alterations and systemic inflammation in a PsA context. These results might be useful for patient stratification and might be considered novel therapeutic targets if validated in larger studies.

In addition, 39 proteins were found significantly increased in SF from PsA patients compared to SF of OA patients. Importantly the most significantly altered molecules were TNFα, IL-17A, IL-6, IL-10, IL-8, ENRAGE, CCL-20, TNFSF-14, OSM, IFNγ, MCP-3, CXCL-11, MCP4, CASP-8, CXCL-6, CD-6, ADA, CXCL-10, TNFβ and IL-7, proteins involved in the immune system process, inflammatory response, cell migration, and cell surface receptor signaling pathway. Among all the altered proteins described in this study, 6 out of 39 have been previously recognized to have a role in the pathogenesis of the PsA and joint inflammation, such as TNFα, IL-6, IL-8, CCL-19, CCL-20, CCL-22 (6–9).

However, along with these, we identified some others that might have a relevant impact on the synovium of PsA, paving the way for new and deeper investigations in this area.

Taken together, our data confirm that synovial fluid proteins can easily be measured by PEA technology and provide new molecular insight into the inflammatory landscape in the synovium in psoriatic arthritis.

## 5 Limitations

This proof of concept highlights the use of PEA technology in the identification of novel proteins or biomarkers in synovial fluid involved in the pathogenesis of PsA. Larger and independent studies would add more value to these findings and might shed light on the implementation of these markers as new tools guiding precision medicine.

## Data availability statement

The original contributions presented in the study are included in the article/supplementary materials, further inquiries can be directed to the corresponding authors.

## Ethics statement

The studies involving human participants were reviewed and approved by ethics committee of the Reina Sofia Hospital. The patients/participants provided their written informed consent to participate in this study.

## Author contributions

All authors listed have made a substantial, direct, and intellectual contribution to the work and approved it for publication.

## References

[1] Belasco J Wei N. Psoriatic arthritis: What is happening at the joint? Rheumatol Ther (2019) 6(3):305–15. doi: 10.1007/s40744-019-0159-1 PMC6702660 31102105

[2] Veale DJ Fearon U. The pathogenesis of psoriatic arthritis. Lancet (2018) 391(10136):2273–84. doi: 10.1016/S0140-6736(18)30830-4 29893226

[3] Lundberg M Eriksson A Tran B Assarsson E Fredriksson S. Homogeneous antibody-based proximity extension assays provide sensitive and specific detection of low-abundant proteins in human blood. Nucleic Acids Res (2011) 39(15):e102. doi: 10.1093/nar/gkr424 21646338 PMC3159481

[4] Arias de la Rosa I López-Montilla MD Román-Rodríguez C Pérez-Sánchez C Gómez-García I López-Medina C. The clinical and molecular cardiometabolic fingerprint of an exploratory psoriatic arthritis cohort is associated with the disease activity and differentially modulated by methotrexate and apremilast. J Internal Med (2022) 291(5):676–93. doi: 10.1111/joim.13447 PMC9310593 35233860

[5] Taylor W Gladman D Helliwell P Marchesoni A Mease P Mielants H. Classification criteria for psoriatic arthritis: Development of new criteria from a large international study. Arthritis Rheumatism (2006) 54(8):2665–73. doi: 10.1002/art.21972 16871531

[6] Altobelli E Angeletti PM Piccolo D De Angelis R. Synovial fluid and serum concentrations of inflammatory markers in rheumatoid arthritis, psoriatic arthritis and osteoarthitis: A systematic review. Curr Rheumatol Rev (2017) 13(3):170–9. doi: 10.2174/1573397113666170427125918 28460627

[7] Mrabet D Laadhar L Sahli H Zouari B Haouet S Makni S. Synovial fluid and serum levels of IL-17, IL-23, and CCL-20 in rheumatoid arthritis and psoriatic arthritis: A Tunisian cross-sectional study. Rheumatol Int (2013) 33(1):265–6. doi: 10.1007/s00296-011-2231-1 22083617

[8] Partsch G Steiner G Leeb BF Dunky A Bröll H Smolen JS. Highly increased levels of tumor necrosis factor-alpha and other proinflammatory cytokines in psoriatic arthritis synovial fluid. J Rheumatol (1997) 24(3):518–23. 9058659

[9] Pickens SR Chamberlain ND Volin MV Pope RM Mandelin AM 2nd Shahrara S. Characterization of CCL19 and CCL21 in rheumatoid arthritis. Arthritis Rheumatism (2011) 63(4):914–22. doi: 10.1002/art.30232 PMC3079365 21225692

[10] Cretu D Prassas I Saraon P Batruch I Gandhi R Diamandis EP. Identification of psoriatic arthritis mediators in synovial fluid by quantitative mass spectrometry. Clin Proteomics (2014) 11(1):27. doi: 10.1186/1559-0275-11-27 25097465 PMC4108225

[11] Leijten E Tao W Pouw J van Kempen T Olde Nordkamp M Balak D. Broad proteomic screen reveals shared serum proteomic signature in patients with psoriatic arthritis and psoriasis without arthritis. Rheumatol (Oxf) (2021) 60(2):751–61. doi: 10.1093/rheumatology/keaa405 PMC7850582 32793974

[12] Struglics A Larsson S Lohmander LS Swärd P. Technical performance of a proximity extension assay inflammation biomarker panel with synovial fluid. Osteoarthritis Cartilage Open (2022) 7; 4(3):100293. doi: 10.1016/j.ocarto.2022.100293 PMC9718077 36474941

